# Emerging zoonoses: A one health challenge

**DOI:** 10.1016/j.eclinm.2020.100300

**Published:** 2020-02-26

December, 2019, marked the third re-emergence in the 21st century of the zoonotic coronavirus (CoV), named severe acute respiratory syndrome corona virus 2 (SARS-CoV-2), crossing species to infect humans. The outbreak of the 2019 novel coronavirus disease (COVID-19), which started in China in the city of Wuhan likely from a wet market of live animals, has caused a rapid increase in the number of infections and deaths across Chinese borders, and has become a serious public and global health threat.

The emergence and re-emergence of zoonotic diseases is not new, and over the past three decades the onset of outbreaks of infectious diseases emerging from animal reservoirs to infect humans has increased. For example, Ebola virus, highly pathogenic avian influenza (HPAI) viruses, and the coronaviruses severe acute respiratory syndrome (SARS) coronavirus and Middle East respiratory syndrome (MERS) coronavirus.

The size of the issue is significant, as epidemiological data by Blancou and colleagues from the University of Athens Medical School (Athens, Greece), reported in *Vet Res* in 2005, show that at least 75% of emerging infectious diseases are zoonotic and originate from wildlife. Prevention activities are difficult to implement because events causing the emerge or re-emerge of zoonoses are complex and affected by multiple factors, such as genetic evolution, demographic changes, environmental conditions, or climate changes affecting the ecosystem.

The unforeseeable onset and rapid dissemination of zoonotic outbreaks means public health systems need to be able to quickly identify early signs of such threats and react promptly. The fact that the beginning of the nCoV-2019 outbreak was observed by at least one physician, as reported by The Guardian on February 2, but not perceived as an urgent threat at the level of decision makers, demonstrates the essential need to identify what could be done better—before new diseases emerge—therefore preventing future outbreaks or, at least, reducing their impact.

To reach this aim, the following steps are crucial: first, the understanding of the causes of disease emergence, the ecology of the agents involved, and their animal hosts; second, the creation of a network able to merge the contributions of different expertise, and work together holistically. At present, the main players of the network are in place (eg, medical doctors, veterinarians, public health experts, and food quality inspectors), but they act separately; for example, veterinarians are not connected with occupational physicians, and in turn they are not in contact with general practitioners who are at the frontline of the disease. Therefore, in the current system, a cohesive network able to receive and act on early warnings at different levels is missing.

The One Health approach is an example of how separate efforts can be aligned to work together effectively. The concept of One Health recognises the interdependence of human health, animal health, and environmental health, and aims to achieve better public health outcomes through the understanding and prevention of risks that originate at the interface between humans, animals, and their environments. Such approach implies a multidisciplinary effort in the implementation of programmes, policies, and research, where multiple sectors communicate and work together, with the common goal of helping disease prediction, prevention, and preparedness.

Three major international organisations, WHO, the UN Food and Agriculture Organization (FAO), and the World Organisation for Animal Health (OIE), have started to put this vision into practice by consolidating a formal partnership to combat human-animal-environment health risks, strengthening their joint action in May, 2018. A year later, as a result of this partnership, they released a guide (Taking a multisectoral, one health approach: a tripartite guide to addressing zoonotic diseases in countries) that provides principles and best practices to assist countries in achieving sustainable and functional collaboration at the human-animal-environment interface.

This effort should be repeated both at the academic and public health level. Rare examples are the University of Washington's Center for One Health Research (Seattle, WA, USA) or the Global Health department at Harvard University (Boston, MA, USA), which has recently promoted the cross-education of its practitioners by collaborations with veterinaries. In public health, interestingly, the US Centers for Disease Control and Prevention is following One Heath strategies against some zoonoses. An example is the response to the Rift Valley Fever (RVF) Virus outbreak in East Africa in 1997. Most of the human infections resulted from direct or indirect contact with the blood or organs of infected animals. To break the chain of transmission, CDC researchers developed a vaccine to vaccinate animals against RVF, therefore preventing its transmission to humans.

Another key area where the One Health approach is needed is monitoring and surveillance. Some professions have greater exposure to zoonotic risks than others—eg, farmers or butchers. Nearly half of the first patients affected by COVID-19 were working at the wet market where the virus originated, according to the first clinical characterisation provided by Chen and colleagues from The University of Hong Kong–Shenzhen Hospital (Shenzhen, China), and published by *The Lancet* in January. Those occupational categories deserve attention as groups at particular risk of zoonotic exposure. In public health departments the collaboration between hygienists, occupational physicians, general practitioners, and veterinarians could provide a push to implement monitoring activities, where the health of workers, animals, and the general population are jointly monitored.

The recent outbreak of the coronavirus infection is a particularly severe example of how close interactions between the health of humans, animals, and the environment can lead to a deadly epidemic. Now it is time to embrace One Health as a framework for public health action against zoonoses, as suggested by the tripartite (WHO, FAO, OIE) zoonotic guide. Governments must prioritise strategies to introduce the One Health approach in education and training programmes and in their public health systems, both at the national and international level. The timely recognition of the interconnection between humans, animals, and environment, intrinsic in the One Health approach, is a key prerequisite for understanding and managing the future of global health threats.

*EClinicalMedicine*

